# Hospital and Institutional News

**Published:** 1921-02-19

**Authors:** 


### HOSPITAL AND INSTITUTIONAL NEWS.

### THE NEW PARLIAMENTARY SESSION.

The new session of Parliament, which was opened by the King in state on Tuesday last, bids fair to be replete with business and discussions of high interest to the readers of these pages. There is the promise, as yet unfulfilled, to bring in reform of the Poor Law. The Ministry of Health has, indeed, an enormous amount of legislative work before it, and the great question arises as to whether the Miscellaneous Provisions Bill will be reintroduced in its old form. Lately a number of arguments have been heard, however, urging the separation of the different proposals, and it seems quite possible that this will be carried out. Then there is the Tuberculosis Bill, introduced last session, which is designed to transfer the exercise of powers from the Insurance Committees to the local authorities, together with a grant of certain additional powers. The Patent Medicines Bill was carried to the Report stage in the House of Lords, and there is also the Dentists Bill, both of which may be introduced to the House of Commons as more or less agreed measures. It is understood that the valuations of the friendly societies for health insurance are proceeding favourably, and that Lord Cave's Committee on Hospital Finance, which is meeting twice a week, can claim good progress in the collection of some very valuable evidence.

### THE PRINCE, F.R.C.S.

On February 14 the Prince of Wales attended the Hunterian Festival Dinner held at the Royal College of Surgeons and was admitted as an Honorary Fellow of the College. The Prince, in a typically happy speech, expressed his appreciation of the honour the College had bestowed upon him and of Surgery, and the great good it had achieved in both peace and war.

### MR. QUENNELL'S RETIREMENT.

The doyen of hospital secretaries, Mr. Sidney M. Quennell, is retiring from the secretaryship of Westminster Hospital at the end of April, thus bringing to a close a record of service as secretary to one hospital which, we believe, is exceeded or even equalled by no other living hospital secretary. Mr. Wilson, whom he followed at Westminster, had held office for forty-five years—from 1832 to 1877—and Mr. Quennell succeeded him in 1878, at the age of thirty-six, having previously been secretary at Poplar Hospital, where his brother-in-law, the late Prebendary Kitto, was honorary secretary. When Mr. Quennell took office the income of Westminster Hospital was £7,895; to-day it is over £50,000; the total expenditure was £11,538, and in 1920 it was £46,000. Mr. Quennell is a convinced upholder of the voluntary system as the essential backbone of our hospitals in the future, supplemented where necessary by certain State assistance, such as, for instance, payment for patients insured under the National Health Acts. Paying patients as such are not taken at Westminster, though since the beginning of this year payments have been received from in-patients, and this source of income brings in at present at the rate of £120 a week; out-patients pay according to a definite scale.

### THE HOUSE COMMITTEE'S RESOLUTION.

The following resolution was passed by the House Committee when Mr. Quennell's resignation was accepted: "That in accepting Mr. Sidney M. Quennell's resignation, after having served in the office of secretary for forty-three years, the House Committee desire to express their great regret at his retirement from a post he has occupied for so lengthy a period with credit to himself and benefit to the hospital. They further desire to place on record their sense of the great value his services have been to the hospital, and the strong claim which such services give him to the liberal consideration of the whole body of Governors." It was also resolved to recommend to the Board of Governors that Mr. Quennell be elected an honorary life governor of the hospital. We hope Mr. Quennell's great experience and wise counsel may be retained, despite his resignation, in a direct way for the benefit of the hospital which he has so devotedly and single-mindedly served. At his age —eighty next birthday—a man is entitled to the fullest ease on retirement, but Mr. Quennell bears so little evidence of his years that it is difficult to contemplate his complete severance from the hospital the furtherance of whose interests have been the constant aim of his long life. His colleagues in the hospital world will join in wishing him many peaceful and happy years to enjoy his well-earned retirement.

### THE STUDENTS' AID TO HOSPITALS.

The help given by the medical students to the London hospitals, a variety, it is true, of the war flag-day collections, has proved infectious, and we note with pleasure that the students of Manchester University spent Shrove Tuesday in a pantomimic procession in aid of the hospitals. Dressed as wild beasts, Mephistopheles, and other old friends, they created both amusement and interest. We hope that the result was worthy of the energy thrown into the plan.

### AN ANTI-TUBERCLE SERUM?

Recently published reports of an anti-serum—or vaccine, it may be—which is said to have solved the problem of tuberculosis treatment are as yet too lacking in detail to justify any optimistic forecasts upon a subject which has already provided the medical profession with so many disappointments. At the moment it is best to realise, with Dr. Leonard Williams, that the whole story of the discovery can, in any case, come out only gradually, admitting that if experiments justify the belief apparently held by men who have used the serum, then a discovery of world-wide importance to humanity has been made.

### DYSENTERY IN BRITAIN.

In a report by Mr. Clifford Dobell, now published by the Medical Research Council, on the occurrence of intestinal protozoon and amœbæ in man, is included the somewhat surprising observation that the amœba of dysentery is relatively common in this country. In certain colliery districts some 7-10 per cent. of the population are infected. There is apparently good reason to suspect that this parasite is, in Great Britain, the cause of more inflammatory conditions than was one time supposed. On the other hand, the reason why infected persons do not in this country develop the acute dysentery of the tropics is possibly that each country develops a special relative immunity against its own race of amœbæ, whereas the stranger in a strange land does not possess this protection. The report is an extremely valuable addition to medical literature, and we hope later to refer to it in greater detail.

### PUBLIC HEALTH IN THE NEAR EAST.

The encouragement of a system whereby the sanitary and health improvement methods of Western Europe may gradually materialise in the Near East is a truly welcome sign. So long has this quarter carried its menace of disease outbreaks that the visit to this country of a medical deputation from Czecho-Slovakia, under the guidance of the Ministry of Health, is an excellent indication of international co-operation. The inspection has covered methods of food storage and supervision, and a considerable programme of visits to important sanitary and other institutions. In the past many such deputations have left this and other countries to learn from the practical experience of their neighbours, and in future the more official encouragement vouchsafed to visits of this type, the better for the health of all.

### THE HOSPITAL BOILER EXPLOSION.

We greatly regret to record that in addition to Mr. J. H. Robinson, the engineer of the Royal Chest Hospital, another death in connection with the recent boiler explosion has taken place, the second victim being Mr. J. J. Swanston, the hall porter. The inquiry by the City Coroner, Dr. Waldo, has been adjourned for the attendance of a Board of Trade expert. We understand that the boiler was an upright hot-water boiler.

### FLAWS IN THE BRIGHTON SCHEME.

The "black-coated worker" has a champion in a correspondent of the *Daily Chronicle*, who thinks that the man earning £350 a year in these days of high rents and railway fares deserves a place in such an insurance scheme as that evolved for Brighton by Dr. Gordon Dill. Such a citizen, especially if he is a family man, cannot afford an operation or a lengthy illness in a nursing home at ten guineas a week, plus the surgeon's fees. Obviously, the correspondent suggests, if the voluntary insurance plan is both to pay the insuring public and to finance the hospitals, it will have to be extended so as to include almost the whole population. The "almost," we presume, is intended to provide for the *nouveau riche*, to treat whom will then be the melancholy and single duty of the consulting physician and surgeon.

### THE BLACK-COATED WORKER.

But there are others who have not forgotten the "black-coated worker," and Mr. Charles A. Parker, F.R.C.S.(Edin.), through the pages of the *Lancet*, suggests that a group or team of medical practitioners should be formed to provide a complete medical service, with a well-equipped health centre, primarily for that large section of the community whose incomes are above the limit of the National Insurance Act, but which is not in a position to provide for itself the costly methods of diagnosis and treatment at the present time. Mr. Parker's scheme is based upon the principles of insurance, which, he considers, having been applied to every other conceivable risk can with equal success be used for medical services. Further, as at present no organised service of consultants and specialists is available to nationally insured persons, it is proposed to throw open such services to them at a modified fee. As regards the main scheme, membership is to be limited to persons having incomes between £250 and £1,500 or £2,000 a year (together with their families). The benefits are most attractive, and include in return for the annual subscription (1) the services of a general practitioner; (2) medical assistance at confinements; (3) consultant and operative services; (4) the same services outside "the team" at specially arranged fees; (5) hospital treatment; and (6) free choice of doctors belonging to the service. We wish we had the space further to describe Mr. Parker's clever and carefully drawn-up scheme, which provides tables showing graduated payments according to income. It is calculated, for instance, that the man with £900 to £1,000 a year will pay a subscription of £7 a year for himself and £2 10s. for each of his dependants. The whole plan deserves close study, and is a distinctly helpful contribution to a subject which many people are keenly interested in at the present time.

### A BARGAIN SALE FOR HOSPITALS.

There are surplus medical stores valued at £500,000 still unsold by the Ministry of Munitions, and by an arrangement concluded with the Joint Council of the Order of St. John of Jerusalem and the British Red Cross Society the Joint Societies will grade and offer these goods for sale on reasonable terms to hospitals, surgical-aid societies, convalescent homes, and institutions of a like nature supported by voluntary contributions. One large depôt will be formed at Shepherd's Bush, where customers can inspect and, if they wish, buy the goods, which will be of two qualities—one perfectly new and the other partly used or soiled in keeping. A circular will be despatched to institutions this month, and inquiries may be addressed to Sir Napier Burnett at the offices of the British Red Cross Society, 19 Berkeley Street, W. In addition to medical stores, the articles for sale will include consignments of hospital beds, bedside tables, blankets, sheets, and other articles in daily use.

This seems to be an excellent opportunity for replenishing equipment, stocks of which have run to a low ebb in many hospitals owing to high prices and shortage of funds. It is understood that the scheme is being carried out at the instance of Lord Inverforth.

### "A MIRACLE AND A GOD-SEND."

Baron Bruno von Schroeder, at last week's meeting of the German Hospital, Dalston, announced that the hospital has received a grant of £12,000 from the National Relief Fund. This sum, he added, which was almost enough to meet the war deficit, was "a miracle and a God-send," and they valued the gift not merely for its money value, but for the spirit in which it had been given. They appreciated, too, the proof contained in the gift of the indispensability of the hospital's work. Some of the grants made by the National Relief Fund have caused heart-searchings, at least on technical grounds, but we are sure that there will be nothing but approval of this one. In sum and in spirit it is worthy of the voluntary system, from which the National Relief Fund was itself ultimately derived.

### SOME EARLY REPORTS.

We are beginning to receive some of the Scottish hospital reports. As Kilmarnock Infirmary, for some undisclosed reason, finishes its financial year on November 11, the secretary and treasurer can get his report out in advance of most people. But it is an excellently prepared statement, although the accounts do not follow the Uniform System; there was a deficit on the year of £305, and legacies amounted to only £21. The advance copy of the statistics of Glasgow Royal Infirmary shows a deficiency of £35,221 as regards ordinary account, which does not include legacies and large donations, amounting together to £42,618, so there was an actual surplus. The working classes contributed £14,368 during the year. The small hospitals are generally the happiest from the financial point of view in these difficult times. The Batley and District Hospital had an income during 1920 of £4,266 (of this £1,268 from workmen's collections) and spent only £4,137. Another small institution, the Pontypool and District Hospital, has produced its full report, well printed, well arranged, and well illustrated, almost within the first month of the year, and with income £8,216 and expenditure £8,013 shows, again, a balance to the good of £202, for there was a comfortable surplus in 1919 also. The Blackburn Royal Infirmary has doubled its income since 1918, greatly helped by the Work-people's Hospital Fund. The ordinary expenditure in the same time has gone up almost in the same proportion, and there being an unusual amount of extraordinary expenditure last year, including extensions to the Nurses' Home, there was an adverse balance of £4,167 on December 31.

### DANGEROUS BALLOONS.

It is only a short time ago, during sundry of the hospital Christmas festivities, that we saw toy gas balloons adding both to the fun and to the decoration of some of the wards, but it is worth while to remember that those filled, to give them buoyancy, with coal gas or hydrogen are by no means devoid of danger. The L.C.C. Committee's recent decision states that "these balloons have been tested in contact with flame and found to explode with a loud report and vivid flash." At entertainments, and indeed in hospitals, light and flimsy decorations may in this way be exposed to considerable risk. For this reason as well as the possible alarm produced it is intended to prohibit the use of these balloons at premises licensed by the Council, and although we do not personally know of any accidents caused by them, the risk is worth consideration by hospital authorities also.

### NEW HOSPITAL FOR GOSPORT.

The general committee of the Gosport War Memorial Committee lately decided to hasten the building of the new hospital at Ann's Hill. Tenders are being invited for a hospital with twenty-two beds: seven for men, eleven for women, and four cots. The money subscribed is nearly £38,000. The site of three acres at Ann's Hill has cost £1,650. Messrs. Young and Hall are the architects, and their original plan for forty beds has been modified, in view of the cost of building, and a first instalment only is being begun. This includes twenty-two beds, an administration block, and a temporary out-patient department. The annual cost of maintaining the hospital is expected to be £3,000. Since the building will absorb most of the money in hand, the committee has recommended an application for a subscription from the Urban District Council. It is hoped by the modifications in the plan to have in hand enough money to form a small endowment for the hospital.

### THIS WEEK'S DRUG MARKET.

The most notable feature is the decided downward movement in the price of cod-liver oil. This is chiefly due to the remarkably good results obtained since the opening of the Western fishing season; the catch of cod has been double what it was at the corresponding date last year, and the same applies to the amount of cod-liver oil yielded. Prices are much lower, and if the good results are maintained throughout the season cod-liver oil should be available for the poor once again. There is a fairly steady demand for eucalyptus oil, and should an impetus be given to this demand prices will probably advance. Lemon oil is offered at comparatively cheap rates. The demand for opium is dull, and prices have a downward tendency. In synthetic products the position is much the same as last week, and offers at below current prices are occasionally met with. Ergot of rye is offered at lower prices. Rhubarb and senna have a lower price tendency. The demand for menthol has been little, and the price has moved upwards to some extent. Liquorice juice is offered at lower rates. Japanese refined camphor has again moved downwards in price. Cream of tartar is cheaper.

